# Supplementary material for: Validation of PozQoL scale in Turkish population living with HIV: a cross-cultural adaptation study
Source: PeerJ. 2024 Aug 21;12:e17873. doi: 10.7717/peerj.17873 (PMC11344534; doi:10.7717/peerj.17873)
Supplement: Supplemental Information 3 [file peerj-12-17873-s003.pdf]

PozQoL Ölçeği

Size sağlığınız, ilişkileriniz ve yaşam memnuniyetiniz hakkında sorular sormak istiyoruz. Lütfen aşağıdaki cümlelerin, sizin için ne sıklıkta geçerli olduğunu 1'den (hiç) 5'e kadar (her zaman) derecelendirerek belirtiniz.

	1 - hiç	2- ara sıra	3 - bazen	4 - çoğunlukla	5 - her zaman
1. Yaşamaktan keyif alıyorum.					
2. Sağlığımla ilgili endişelerim var.					
3. Çevremdeki insanlara karşı aidiyet hissetmiyorum.					
4. HIV pozitif olmamın, istediğimi yapmamı engellediğini hissediyorum.					
5. Kişisel olarak kendimi iyi hissediyorum.					
6. HIV yaşamımda karşıma çıkan fırsatları kısıtlıyor.					
7. HIV'in sağlığım üzerindeki etkileri konusunda endişeliyim.					
8. Hayatımın kontrolünün bende olduğunu hissediyorum.					
9. HIV taşıdığımı öğrendiklerinde, insanların beni reddetmesinden korkuyorum.					
10. HIV ile başa çıkmaya çalışmak beni yoruyor.					
11. HIV'in kişisel ilişkilerimi kısıtladığını hissediyorum.					
12. Yaşlandığımda HIV'in sağlığım üzerinde yapacağı etkilerden korkuyorum.					
13. Geleceğime dair iyimserim.					
